# Assessing Variability in Vascular Response to Cocoa With Personal Devices: A Series of Double-Blind Randomized Crossover n-of-1 Trials

**DOI:** 10.3389/fnut.2022.886597

**Published:** 2022-06-13

**Authors:** Mariam Bapir, Paola Campagnolo, Ana Rodriguez-Mateos, Simon S. Skene, Christian Heiss

**Affiliations:** ^1^Department of Clinical and Experimental Medicine, Faculty of Health and Medical Sciences, University of Surrey, Guildford, United Kingdom; ^2^Department of Biochemical Sciences, Faculty of Health and Medical Sciences, University of Surrey, Guildford, United Kingdom; ^3^Department of Nutritional Sciences, School of Life Course and Population Sciences, Faculty of Life Sciences and Medicine, King’s College London, London, United Kingdom; ^4^Vascular Department, Surrey and Sussex NHS Healthcare Trust, Redhill, United Kingdom

**Keywords:** cocoa flavanols, n-of-1, blood pressure, pulse wave velocity, variability, flow-mediated dilation

## Abstract

Controlled clinical intervention studies have demonstrated that cocoa flavanols (CF) can decrease blood pressure and arterial stiffness in healthy humans, although a large variability in the effect size across trials has been reported. In this study, we evaluated the intra- and inter-individual variability of responses to CF in everyday life using a series of n-of-1 trials in healthy free-living individuals with normal blood pressure carrying personal devices. In total, eleven healthy young humans participated in a repeated crossover randomized controlled double-blind n-of-1 trial. On 8 consecutive days, each volunteer consumed on alternating days 6 CF capsules (862 mg CF) on 4 days and 6 matched placebo capsules (P, 0 mg CF/day) on another 4 days in one of the two randomized sequences (CF-P-CF-P-CF-P-CF-P or P-CF-P-CF-P-CF-P-CF). On each day, the capsules were taken at the same time in the morning with breakfast after baseline measurements. Each subject was provided with an upper arm blood pressure monitor and a finger clip that measures pulse wave velocity (PWV). Measurements of blood pressure, heart rate, and PWV were taken at least hourly over 12 h during the day by the participants. On the first 2 days, measurements were performed under supervision to provide training. The overall mixed model analysis showed that CF significantly decreased 12-h systolic blood pressure and PWV by −1.4 ± 0.3 mmHg and −0.11 ± 0.03 m/s, respectively. Peak effects were observed within the first 3 h (1.5 h SBP: −4.9 ± 2.2 mmHg, PWV: −0.32 ± 0.17 m/s) and again after 8 h post-ingestion. Large inter-individual variation in responses was found [intra-cluster correlation coefficients (ICC): 0.41, 0.41]. When analyzing single individuals’ datasets, there was also considerable between-day variation in individual responses that varied greatly between subjects (ICC: 0–0.30, 0–0.22, 0–0.45). Effect sizes inversely correlated with baseline blood pressure values both between- and within-subjects. The data confirm that cocoa can decrease blood pressure and arterial stiffness in everyday life when elevated within the normal range. The large inter- and intra-individual variation in responses calls for more personalized nutritional intervention strategies.

## Introduction

Epidemiological data suggest that there is an inverse relationship between the consumption of flavonoid-rich diets and the risk for cardiovascular disease (1–6). Significant associations were also observed for one subclass of flavonoids, the flavanols, and their oligomers, the procyanidins (2, 7). Dietary intervention studies with flavanol and procyanidin-containing foods support this notion, as the consumption of these foods improves arterial endothelial function while lowering blood pressure and arterial stiffness in healthy subjects (8–10). However, the clinical intervention studies were performed in tightly controlled experimental settings, and in particular, effects on blood pressure show large heterogeneity between trials and suggest that baseline blood pressure may play a role in the effect of cocoa on blood pressure (10), As participants’ responses were only measured once at specified times after single or repeated ingestion of interventions in the laboratory, individual responses were not investigated at different days and not during real life. A profound understanding of inter- and intra-individual variability of responses in real life is an important area of research in a world that aims at personalized nutrition and medicine.

In the so-called n-of-1 study design, individuals are exposed to interventions and control multiple times (11, 12). It has been proposed recently as a powerful tool allowing to study small effects even in fewer subjects as inter-individual variability is reduced by repeatedly performing interventions in the same individuals. More importantly, it allows to study the variability of responses in individual people to identify “responders” and “non-responders” and also to understand determinants of the response (13). Some authors have argued that n-of-1 trials may be the ultimate strategy for individualizing medicine (13). The overall aim of this study was to evaluate the intra- and inter-individual variability in vascular responses to cocoa flavanols using personal devices and n-of-1 study design.

## Materials and Methods

### Study Design and Participants

The study participants (*n* = 11) were healthy male and female adult subjects (<45 years) with normal blood pressure and without history, signs, or symptoms indicative of cardiovascular disease, including previous myocardial infarction, stroke, and peripheral artery disease or current or previous medication.

**FIGURE 1:**
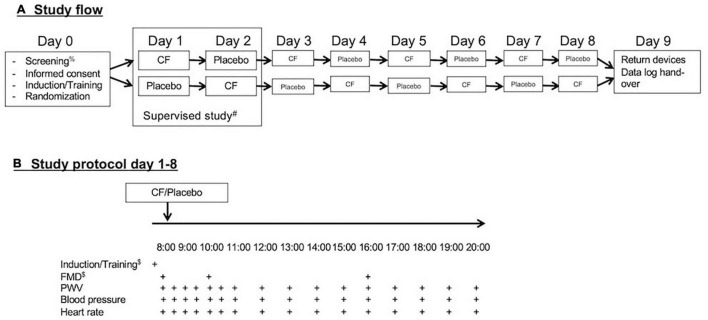
**(A)** Study flow and **(B)** study protocol. After screening for eligibility and obtaining signed informed consent on day 0, participants received training and were randomized. They performed the first two study days under supervision and the remaining 6 days independently via self-monitoring with personal devices during everyday life. After completion of the eight study days, the participants return their devices and submitted records of reading.

This study was designed as an exploratory repeated, randomized, controlled crossover study. With personal devices provided by the investigators (blood pressure cuff and fingerclip), the study participants self-monitored blood pressure, heart rate, and pulse wave velocity (PWV) over 12 h during the day on 8 days (Figure 1). Measurements were taken immediately before and every 30 min over the first 3 h after consumption of capsules and at hourly intervals for a total of 12 h.

On each of the 8 study days, participants consumed either 6 cocoa flavanol capsules (containing a total of 862 mg cocoa flavanols) or 6 placebo (empty 00 capsules filled with brown sugar). All participants were randomized to one of the two different sequences of treatment periods. The alternating treatment allocations were either flavanol-placebo-flavanol-placebo-flavanol-placebo-flavanol-placebo or placebo-flavanol-placebo-flavanol-placebo-flavanol-placebo-flavanol. All capsules were provided in black opaque zip-lock bags labeled with an alphanumeric code, and capsules were supposed to be directly tipped into the mouth and swallowed with water together with breakfast.

On the first 2 days of the study, measurements were performed under supervision at the universities research facility to provide training to participants. On the following days, the measurements were performed during the normal life of the participants. The participants were instructed to sit down for 10 min before measurements and take measurements of blood pressure, heart rate, and PWV on the left arm with the arm resting on a table in front of them. They were also taught how to use the software to operate the devices on a provided small tablet device (iPod touch, Apple, United States) and introduced to an electronic case report file to which participants transferred their data.

On the training days of 8 subjects, we also performed measurements of flow-mediated dilation (FMD) at baseline before (0 h), at 2 h, and at 8 h after the first intervention to ensure the biological activity of test materials. The allocation was double-blinded and randomized, and the randomization sequence was generated using an online calculator^1^. The study protocol was reviewed and approved by the University of Surrey Ethics Committee with the registration number FER-1819-040, and written informed consent was obtained from all participants.

### Cocoa Interventions and Placebo

The cocoa supplement and placebo capsules were purchased from Amazon.co.uk. According to the provided supplement facts, 6 capsules of CocoaVia supplement (Mars Inc., United States) contained 862 mg (95%CI: 849, 875 mg) CFs. CFs as defined here correspond to the sum of flavanol monomers and procyanidins with a degree of polymerization up to 7, according to the AOAC 2020.05 method (14). While cocoa flavanols in the supplement were initially quantified with a previously validated method, conversion to current AOAC2020.05 method reporting standards was done using a published conversion method (15). Importantly, reporting of (-)-epicatechin content remained unaffected when applying this conversion method. In addition, actual CF and (-)-epicatechin content in the supplement remained unchanged throughout the trial (15, 16). Placebo capsules were identical size empty capsules (YourSupplement Gelatin Capsules, Size 00, United Kingdom) that were filled with brown sugar. The composition is detailed in Table 1. As described earlier, capsules were consumed after baseline measurements in the morning together with breakfast. All capsules were provided to participants in black non-transparent zip-lock bags.

**TABLE 1:** Composition of capsules.

	Cocoa flavanols	Placebo
Number of capsules (n)	6	6
Cocoa extract (mg)	3210	0
Total cocoa flavanols (mg)	862	0
(-)-Epicatechin (mg)	160	0
Caffeine (mg)	40	0
Theobromine (mg)	150	0
Energy (kcal)	7	10
Carbohydrates (g)	2	3

### Measurement of Blood Pressure, Heart Rate, and Pulse Wave Velocity

Standard upper arm systolic and diastolic blood pressure and heart rate (OMRON Evolv, Milton Keynes, United Kingdom) and finger PWV (iHeart device, VitalSines International, Vancouver, BC, Canada) were measured in 30 min intervals for the first 3 h after the morning intake of the capsules and at hourly intervals over a total of 12 h (see Figure 1). The cuff size was tested during training sessions and was appropriate for all participants. The participants were asked to rest in a sitting position for 5 min prior to seated measurements with the non-dominant arm resting at heart level. Each measurement was to be performed in triplicate. The first measurement was discarded, and the average of the two remaining measurements was used for statistical analyses. The values were recorded on respective applications on an iPod touch and Excel spreadsheets both provided to participants. Participants were also instructed to refrain from taking other dietary supplements, perform exercise training, and avoid alcohol (>1 unit) on the 1 day prior to study days and the study days. The average deviation between the 2 measurements was for SBP −0.6 mmHg (range: −2.3 to 0.9 mmHg), for DBP 0.7 mmHg (range: −0.7 to 1.8 mmHg), for heart rate −1.9/min (range: −4.8 to 0.5/min), and PWV −0.1 m/s (range: −1.0 to 0.3 mmHg). The standard deviations of average deviations differed between study participants. The ranges for SDs were (SBP: 3–6 mmHg, DBP: 2–7 mmHg, heart rate: 2–5/min, and PWV: 0.2–1.0 m/s).

### Flow-Mediated Dilation

All of our previous studies have confirmed positive responses to cocoa flavanols on FMD as primary end point, while only some but not others showed significant effects on blood pressure (8, 9, 17). However, FMD is difficult to measure during real life. Therefore, and to ensure that the interventions possess biological activity, we measured FMD on participants only on the first 2 days during the supervised training. Due to the alternating allocation of CF and placebo, each participant received measurements at baseline before (0 h), at 2 h, and at 8 h after the first CF and first placebo intervention. FMD measurements were only introduced after 3 people had already completed their n-of-1 trial as it became clear that the variability of blood pressure responses was greater than expected.

The FMD was measured as previously described (18). Briefly, the diameter and flow velocity of the brachial artery (BA) were measured using a 12 MHz transducer (Vivid I, GE) and automatic edge-detection software (Brachial Analyzer, Medical Imaging Applications, Iowa City, IA, United States) yielding standard deviations of mean differences between repeated measurements of less than 1%. BA diameter was measured at 2 cm proximal to the elbow. Reactive hyperemia was induced by 5 min of lower arm occlusion with a sphygmomanometer cuff inflated to 200 mmHg. Images were recorded continuously (Vascular Imager, Medical Imaging Applications, Iowa City, IA, United States) over 10 s for baseline, over within the last 30 s of occlusion (10 s recording), and over 3 min after cuff deflation. FMD was calculated as the maximal relative diameter gain relative to baseline. The FMD was expressed as (diameter_max_-diameter_baseline_)/diameter_baseline_ × 100.

### Statistical Analyses

Characteristics of the study population are presented as mean and standard deviation. For each parameter [systolic and diastolic blood pressure (SBP and DBP), heart rate (HR), and pulse wave velocity (PWV)], a fitted model was applied that adjusts for the baseline measure of the parameters on each study day and includes an intervention effect, time, and an interaction between time and intervention to allow for the effect to change with time. The analysis was performed first with all values over 12 h included and then repeated on a restricted dataset only including baseline and 1.5 h values. The statistical models were separately fitted for the group and for individuals (*n* = 1). A random subject effect accommodates the correlation between the repeated measurements in day/time within-subjects and is substituted by a random day effect in the individual models. By using the intraclass correlation coefficient (ICC), a ratio of the within-individual variance to the total, the inter-individual reliability of a measure can be indicated. High ICCs indicate that variation in measures is attributable to differences between individuals and is not dominated by day-to-day variation within individuals [see, for example, (19)].

## Results

The characteristics of the study population are summarized in Table 2. The supplement was well tolerated by 10 of the participants. One participant discontinued after 5 days (3 CF, 2 placebo) due to problems in swallowing the capsules and upset stomach, but all observations of *n* = 11 contribute to the mixed model analysis.

**TABLE 2:** Baseline characteristics of the study population (values are mean and standard deviation and range).

*N*		11		
Sex (male/female)		3/8		
Age (years)	24	±	2	(20–44)
Weight (kg)	66	±	13	(53–95)
Height (kg)	1.72	±	0.10	(1.61–1.92)
Body mass index (kg/m^2^)	22.0	±	4.0	(17–28)
Ex-smoker (n)		2		
Systolic blood pressure (mmHg)	115	±	8	(100–127)
Diastolic blood pressure (mmHg)	74	±	7	(65–85)
Heart rate (/min)	73	±	12	(60–82)
Pulse wave velocity (m/s)	8.2	±	0.9	(7.1–9.4)
Flow-mediated dilation (%)	7.0	±	1.3	(5.9–9.9)

**FIGURE 2:**
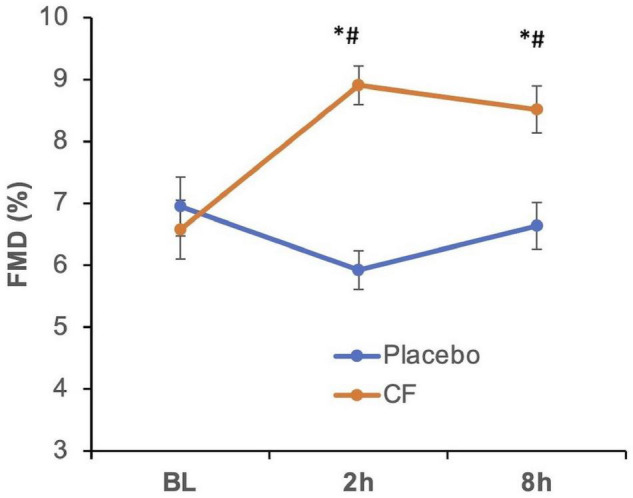
Effect of cocoa flavanols (CF, orange line) as compared with placebo (blue) on flow-mediated dilation (FMD) in healthy participants at baseline (BL) before cocoa flavanol or placebo (blue line) ingestion and at 2 and 8 h thereafter. Overall, *p* < 0.05 (two-way repeated measurements ANOVA), *p < 0.05 vs. BL, *#p* < 0.05 vs. respective placebo timepoint.

The ingestion of CF supplement but not placebo resulted in a significant increase of FMD values compared with baseline and placebo at 2 and 8 h (see Figure 2) confirming the biological activity of the used CF supplement. Supplementary Figure 1 shows the CF-related responses of the individual participants.

### Analysis of Overall Effects

A first step was to evaluate the overall effects (SBP, DBP, HR, PWV) in a way that would allow comparison with other conventional studies in which participants’ measurements are taken only at selected timepoints in each study arm once for each intervention and placebo.

**FIGURE 3:**
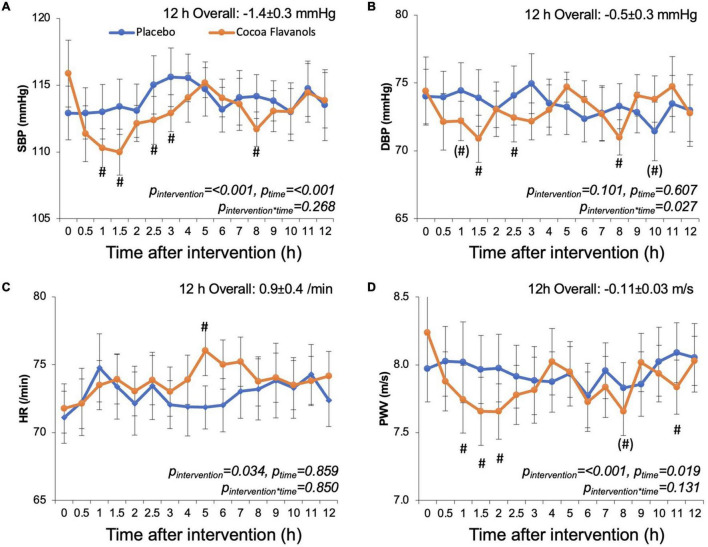
Effect of cocoa flavanols (orange) or placebo (blue) on time courses of **(A)** systolic blood pressure (SBP), **(B)** diastolic blood pressure (DBP), **(C)** heart rate (HR), and **(D)** pulse wave velocity (PWV). Cocoa flavanols and placebo capsules were ingested after 0 h baseline measurements. Values are average values and standard error from the average values obtained over 12 h in each of the *n* = 11 on the 4 cocoa flavanol days and 4 placebo days. *p*-values are based on a statistical mixed model adjusting for baseline measure, including intervention time and intervention × time interaction effects, #*p* < 0.05 and (#) *p* < 0.1 vs. respective placebo timepoint.

Figure 3 shows the averaged timecourse of blood pressure, heart rate, and PWV over 12 h. Supplementary Figure 2 shows exemplary original raw data (SBP, DBP, HR, PWV) from one of the participants. The overall mixed model analysis integrating all data showed that CF significantly decreased systolic blood pressure and PWV by −1.4 ± 0.3 mmHg (mean ± SE; *p_*intervention*_* < 0.0001, *p_*time*_* < 0.001, p_interaction intervention*time_ = 0.268) and −0.11 ± 0.03 m/s (*p_*intervention*_* = ≤ 0.0001, *p_*time*_* = 0.019, *p_*interaction*_* = 0.131), respectively. There was also a decrease in diastolic blood pressure that depended on time (−0.5 ± 0.3 mmHg) (*p_*intervention*_* = 0.101, *p_*time*_* = 0.607, *p_*interaction*_* = 0.027). The heart rate significantly increased by 0.9 ± 0.4/min (*p* < 0.034, *p_*time*_* = 0.859, *p_*interaction*_* = 0.850). The responses greatly varied between participants as indicated by intra-cluster correlation coefficients (ICC) (0.41, 0.24, 0.39, 0.41). The variation in responses did not differ between the first 2 supervised and the remaining 6 home-based unsupervised measurements. Using the consecutive study days as a continuous covariate, we found no evidence that over the course of 8 days, there was a cumulative effect of CF despite interspersed placebo days.

The time courses (Figure 3) were qualitatively similar in terms of SBP, DBP, and PWV with significant CF-related decreases within the first 3 h. While values returned to baseline, we observed a second somewhat smaller phase of CF-related SBP, DBP, and PWV decreases at around 8 h. The overall time course of changes in heart rate indicated a peak at around 5 h.

We then evaluated the magnitude and inter-individual variation of peak effects at 1.5 h after ingestion. This analysis showed greater effects as compared with the overall 12 h effects. SBP and PWV decreased by −4.9 ± 2.5 mmHg (*p_*intervention*_* = 0.032, *p_*day*_* = 0.645, *p_*interaction*_* = 0.702). In this restricted dataset, the decrease in PWV did not reach statistical significance (−0.31 ± 1.7 m/s, *p_*intervention*_* = 0.060, *p_*day*_* = 0.269, *p_*interaction*_* = 0.374). A numerical decrease in DBP was observed that was not significant (−2.4 ± 1.6 mmHg, *p_*intervention*_* = 0.139, *p_*day*_* = 0.840, *p_*interaction*_* = 0.768). There was a significant time-dependent decrease in heart rate −0.5 ± 1.5/min (*p_*intervention*_* = 0.745, *p_*day*_* = 0.041, *p_*interaction*_* = 0.065). The responses on the level of SBP, DBP, and PWV at 1.5 h much less varied between participants as compared with 12 h effects as indicated by low ICC (0.01, 0.10, 0.11), while ICC of heart rate was 0.59 rather indicating large between-subject variation.

### Inter- and Intra-Individual Variation of Blood Pressure, Heart Rate, and Pulse Wave Velocity Responses

**FIGURE 4:**
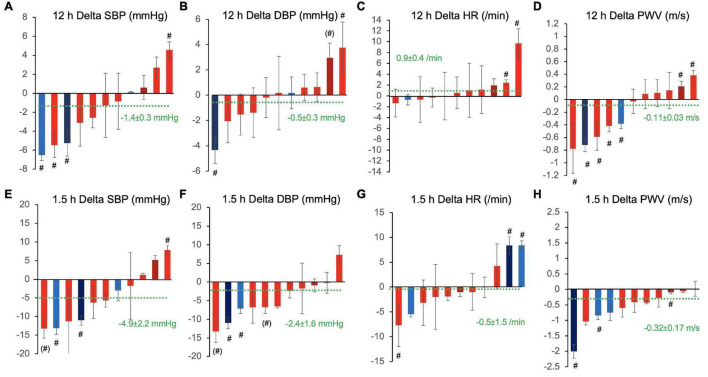
Inter-individual variation of 12 h overall **(A–D)** and 1.5 h peak responses **(E–H)** to cocoa flavanols as compared with placebo in terms of **(A,E)** systolic blood pressure (SBP), **(B,F)** diastolic blood pressure (DBP), **(C,G)** heart rate (HR), and **(D,H)** pulse wave velocity (PWV). Waterfall diagrams showing individual average effect in each of the 11 study participants sorted by magnitude of effects. Columns are mean effects and standard error. Green dotted line indicates the overall effect including all individuals (*n* = 11). #*p* < 0.05 and (#) *p* < 0.1, individual effects of cocoa flavanol intervention vs. placebo. Red shade designates female participants, blue shade designates male participants, and darker shade indicates ex-smokers.

To further evaluate the variability of responses, we fitted statistical models to each of the 11 subjects’ dataset individually. Figure 4 shows the 12 h overall and peak 1.5 h CF responses over placebo of each participant in waterfall diagrams ordered by the magnitude of effects. The magnitude of responses greatly differed between individuals with some showing clinically quite relevant decreases of blood pressure in the order of –5 to 10 mmHg and some even showing unexpected inverse responses with statistically significant blood pressure increases in particular when evaluating the 1.5 h responses.

**FIGURE 5:**
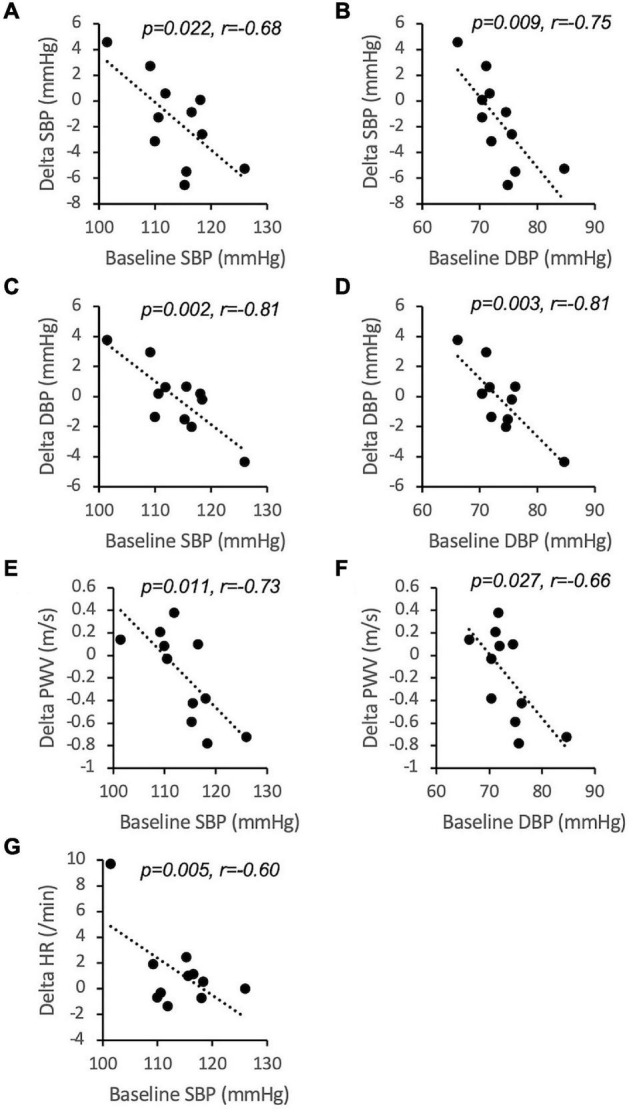
Inverse correlations between baseline values of systolic blood pressure (SBP, **A,C,E,G**) and diastolic blood pressure (DBP, **B,D,F**) and 12 h overall inter-individual responses to cocoa flavanols over placebo (*n* = 11). *r* is Pearson’s correlation coefficient. PWV, pulse wave velocity; HR, heart rate.

In an attempt to identify determinants of inter-individual variability, we tested if significant correlations existed between the overall effect of CF on SBP, DBP, HR, and PWV and characteristics at baseline. There were no significant correlations between age, height, weight, BMI and FMD. Interestingly, the CF-related change in SBP, DBP, and PWV inversely correlated with baseline SBP and DBP (Figure 5). The change in heart rate was inversely correlated with baseline SBP.

The 12 h response to CF varied also between days within participants, and the degree of variation differed between participants as indicated by wide ranges of individual ICC value (SBP: 0–0.30; DBP: 0–0.22; HR: 0–0.67; PWV: 0–0.45).

**FIGURE 6:**
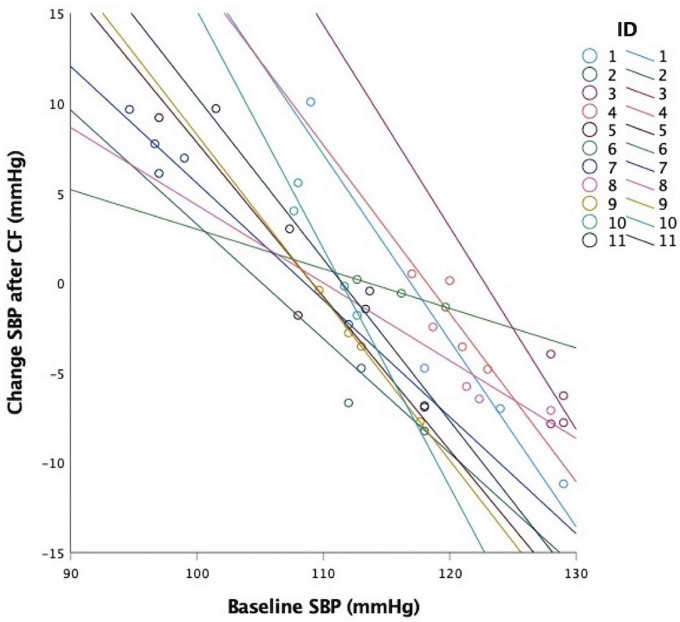
Dependence of intra-individual systolic blood pressure (SBP) responses on baseline SBP before CF intervention. Four individual CF 12 h overall responses of each of the *n* = 11 participants (each color represents one participant).

To illustrate this, Figure 6 shows baseline SBP and before and overall change of SBP after CF ingestion over 12 h (4 data points from *n* = 10 and 3 data points from *n* = 1). Note that all 11 show a linear fit line with negative slopes and that several participants showed both positive and negative responses depending on the baseline value in the morning before CF ingestion. This indicates that CF did not lower blood pressure when values were already low (approximately below 115/75 mmHg).

## Discussion

This study shows for the first time a significant real-life biphasic response in terms of blood pressure and arterial stiffness lowering with maximal effects within 3 h and later at approximately 8 h. This was paralleled by increases in FMD at 2 and 8 h. The improvement of FMD by approximately 2% at 2 h was similar to what was obtained in other previous studies in healthy volunteers using slightly different cocoa flavanol interventions (9, 20, 21). The time-dependent effect of cocoa flavanols has been studied many times over the first 5–6 h after single acute ingestion and after daily consumption over days and weeks. Most of the acute studies show that peak FMD improvements occur at 1–2 h after single ingestion and return to baseline after 3 h, and daily consumption over weeks leads to a chronic improvement of FMD that lasts at least for 24 h after the last ingestion (9, 20–24). The significant increase in FMD (and decrease in blood pressure and PWV) at 8 h observed in this study has not been described previously and has interesting implications. Most studies that have performed kinetic examinations of acute single ingestion FMD effects were up to 6 h only. In these studies, FMD improved over the first 3 h with a peak effect at 1–2 h and returned to baseline by 5–6 h (17, 22). These effects may be linked to the absorption and bioavailability of structurally related epicatechin metabolites (23). However, newer literature shows that at later time points, a large proportion of cocoa flavanols that are not absorbed in the upper intestine will undergo metabolism by large intestinal microbiota that occur later in blood (25, 26). The fact that no blood samples were taken is a limitation of this study as the analysis of plasma flavanol metabolites could have provided correlative evidence in support of the gut metabolites hypothesis.

Alongside the overall analysis of the results, this study investigates for the first time intra- and inter-individual responses to cocoa flavanols using the n-of-1 study design. It shows that a large proportion of intra- and inter-individual variation effects were related to baseline blood pressure values. Not all participants responded and not all responded all the time. Specifically, if the blood pressure was low in a person on a certain day, the cocoa flavanols decreased blood pressure and arterial stiffness less. This has important safety and mechanistic implications. In particular, in primary prevention, safety is a major issue, and excessive blood pressure lowering may pose a safety risk as described for antihypertensive medication (27) for the potential use of flavanols in primary prevention in low-risk individuals without risk factors and normal blood pressure (28). The magnitude of effects in particular within the first 3 h is similar to what standard antihypertensive medications achieve in clinical trials (29), highlighting the clinical relevance and potential of flavanols for use in clinical practice. Whether or not a continuous decrease in blood pressure can be achieved with repetitive administration remains to be investigated. The increase in heart rate we have observed at around 5 h may be due to the high theobromine content of the cocoa supplement and needs to be taken into account as a potential side effect (30) that could also mask blood pressure lowering effects of CF. In particular in young and healthy people, an increase in heart rate is a physiological response to peripheral vasodilation and can even prevent blood pressure lowering of antihypertensive drugs like calcium channel blockers (amlodipine) (31). This is also why calcium channel blockers exert more effective blood pressure lowering when combined with beta blockers that prevent compensatory increase in heart rate (32). In line with our findings, the results from previous studies investigating blood pressure effects of cocoa flavanols were less consistent than studies investigating FMD (33). The mechanisms of how cocoa flavanols improve vascular function are not entirely understood beyond the knowledge that nitric oxide bioavailability is likely causally involved in acute effects (23), and long-term effects, which are less comparable with this study, are linked to changes in gene expression profiles (34). Whether the same mechanisms mediate effects on endothelial function, blood pressure, and arterial stiffness is not known. Of note, gene expression changes that correlated with changes in FMD significantly differed from those associated with blood pressure and PWV changes (34). One may argue that this study supports that cocoa flavanols rather improve vascular homeostasis than being direct vasoactive molecules as the latter would be expected to lower blood pressure more and even if low at baseline.

The variability of responses highlights the importance of personal devices that can monitor biomarkers of biological effects frequently as therapeutic targets for personalized medicine. For instance, home blood pressure monitoring is now recommended in clinical practice guidelines (28). Our data clearly demonstrate that people respond differently to identical treatments and even one person may respond differently on different days. Wearable devices to measure the responses frequently or even continuously will be an important tool to understand individual responses and gauge individual treatments effectively. The devices used in this study required manual activation of measurements that limits wider implementation. The development of automated non-invasive devices for measuring blood pressure (35) without the need for a cuff inflation will be important to avoid interruption of normal life or sleep.

The effective exploitation of n-of-1 trials for personalized medicine requires standardized statistical models to analyze the results, and there is currently no standard approach. In this study, we adopted mixed model approaches that allow adjustments for baseline measures of the parameters on each day and include an intervention effect, time, and an interaction between time and intervention, as well as random subject or day effects to appropriately capture the between- and within-subject variation. The ability to compare the pooled analysis over subjects and study individual effects demonstrates the importance of further investigation of individualized approaches over simple population-based methods.

Several limitations not previously mentioned apply to this study. The small sample size limits generalizability to the wider population. Furthermore, the effect of CFs may be influenced by the diet and, in particular, the breakfast with which it was consumed. Unfortunately, we did not collect data related to the diet consumed throughout the study or take blood samples to evaluate biomarkers of polyphenol intake to investigate this. Another limitation relates to the fact that the cocoa supplement did not only contain cocoa flavanols but also significant amounts of methylxanthines, primarily theobromine. It is now appreciated that methylxanthines can have health effects on their own (36). The placebo in this study did not contain any methylxanthines. However, we have previously demonstrated that methylxanthines at up to 200 mg do not exert significant effects on endothelial function, blood pressure, and arterial stiffness in healthy young volunteers but rather increase the bioavailability of flavanols over the first 5 h (21). Therefore, it is reasonable to assume that most effects with the exception of heart rate in this study are driven by the flavanols. In addition, we did not test endothelium-independent vasodilation in response to nitroglycerin which is often part of the vascular assessment protocol (37). Therefore, endothelium independent effects cannot be excluded, but are rather unlikely as a number of previous studies did not show any effects on nitroglycerin-mediated dilation in healthy populations (8, 9). Finally, we need to point out that effects on PWV are unlikely to be due to short-term effects on arterial structural stiffness but are most likely secondary to blood pressure lowering effects. Finally, the amounts of CF [862 mg total, 160 mg (-)-epicatechin] applied in this study are relatively high but are in the range that can be achieved with regular diet. The mean daily intake of total flavanols in central Europe was estimated based on questionnaires at 449 mg [13–14 mg (-)-epicatechin] (38, 39). A more recent study indicates a steep dose response between all-cause, cardiovascular and cancer mortality, and flavanol monomer intake up to approximately 100 mg/day and then plateaus (2). The daily flavanol monomer intake reported in this study ranged from 0 to 916 mg (oligo + polymers: 0–2,254 mg) (2). The dose to achieve half maximal effects in humans (ED50) in terms of acute endothelial functional increases at 2 h after single ingestion is approximately 0.5 mg (-)-epicatechin/kg [body weight] in healthy humans with maximal effects achieved at *ca.* 2 mg/kg (21, 40). The dose response in terms of blood pressure decreases is not well established but may require higher amounts (41). Therefore, the amounts administered were chosen as they are in the range that can be achieved with normal diet and would be expected to maximally improve endothelial function. They are similar to what was used in the recent COSMOS trial (500 mg CF/day) that showed a significant reduction in cardiovascular mortality when consumed over several years (42).

In conclusion, our data confirm that cocoa can improve vascular function and decrease blood pressure and arterial stiffness not only within the first 3 h after ingestion but also later at 8 h in healthy normotensive people. Our study uncovers considerable intra- and inter-individual variation in responses using an innovative n-of-1 study design with personal devices. With this approach, we uncovered baseline blood pressure as a major determinant of response in healthy young people. Our data highlight the need for personal health monitors to develop and implement effective personalized nutritional intervention strategies in the future.

## Data Availability Statement

The original contributions presented in this study are included in the article/Supplementary Material, further inquiries can be directed to the corresponding author.

## Ethics Statement

The studies involving human participants were reviewed and approved by the University of Surrey Ethics Committee. The patients/participants provided their written informed consent to participate in this study.

## Author Contributions

MB: project administration and preparation, investigation, analysis, and writing – review and editing. PC: supervision and review and editing. AR-M: review and editing. SS: statistical analysis and review and editing. CH: conceptualization, project administration, supervision, investigation, analysis, and writing – review and editing. All authors contributed to the article and approved the submitted version.

## Conflict of Interest

The authors declare that the research was conducted in the absence of any commercial or financial relationships that could be construed as a potential conflict of interest.

## Publisher’s Note

All claims expressed in this article are solely those of the authors and do not necessarily represent those of their affiliated organizations, or those of the publisher, the editors and the reviewers. Any product that may be evaluated in this article, or claim that may be made by its manufacturer, is not guaranteed or endorsed by the publisher.

## Footnotes

1
https://www.graphpad.com/quickcalcs/randomize1/
