# Supplementary material for: Assessing Variability in Vascular Response to Cocoa With Personal Devices: A Series of Double-Blind Randomized Crossover n-of-1 Trials
Source: Front Nutr. 2022 Jun 13;9:886597. doi: 10.3389/fnut.2022.886597 (PMC9234529; doi:10.3389/fnut.2022.886597)
Supplement: Supplementary file 1 [file Data_Sheet_1.docx]

**SUPPLEMENTAL FIGURES**

Assessing variability in vascular response to cocoa with personal devices: a series of double-blind randomized cross-over n-of-1 trials

Mariam Bapir^1^, Paola Campagnolo^2^, Ana Rodriguez-Mateos^3^, Simon S. Skene^1^, Christian Heiss^1,4^

^1^ Department of Clinical and Experimental Medicine, University of Surrey, Faculty of Health and Medical Sciences, Guildford, United Kingdom

^2^ Department of Biochemical Sciences, University of Surrey, Faculty of Health and Medical Sciences, Guildford, United Kingdom

^3^ Department of Nutritional Sciences, School of Life Course and Population Sciences, Faculty of Life Sciences and Medicine, King’s College London, London, United Kingdom

^4^ Vascular Department, Surrey and Sussex NHS Healthcare Trust, Redhill, United Kingdom

*** Correspondence:**Prof. Dr. Christian Heiss, University of Surrey, Faculty of Health and Medical Sciences, Stag Hill, Guildford GU2 7XH, United Kingdom. Phone: +44 7878589817, Email: c.heiss@surrey.ac.uk.

**Supplemental figure legends**

**Supplemental Figure 1:** Individual flow-mediate dilation (FMD) responses of 8 participants in order of effect size. Values represent the difference between changes (2h minus baseline) on the first placebo and cocoa flavanol day (Delta cocoa flavanol minus placebo). Red colour designates females, blue males and darker shades indicate ex-smokers. Green dotted line indicates average effect (2.5%).

**Supplemental Figure 2:** Exemplary timecourse of systolic and diastolic blood pressure (SBP, DBP) and pulse wave velocity (PWV) of one participant during the entire study duration of 8 days. Displayed on x-axis are hours post ingestion of the cocoa flavanol (CF) and placebo (P) capsules.


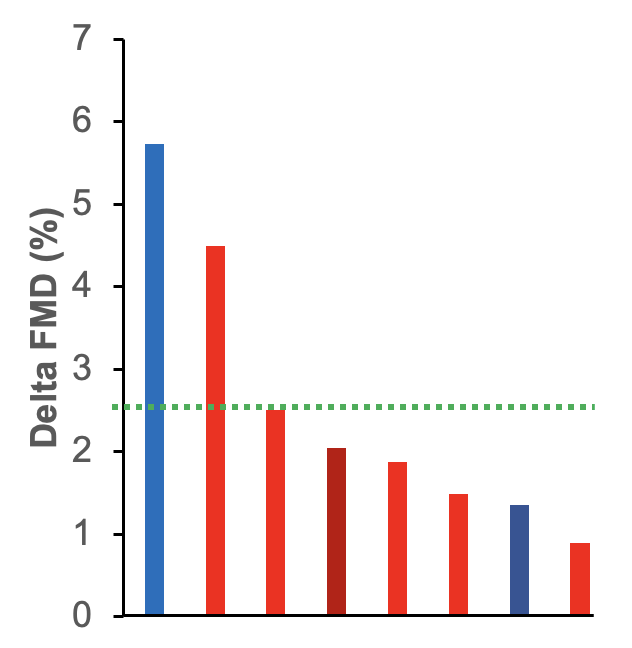


**Supplemental Figure 1**


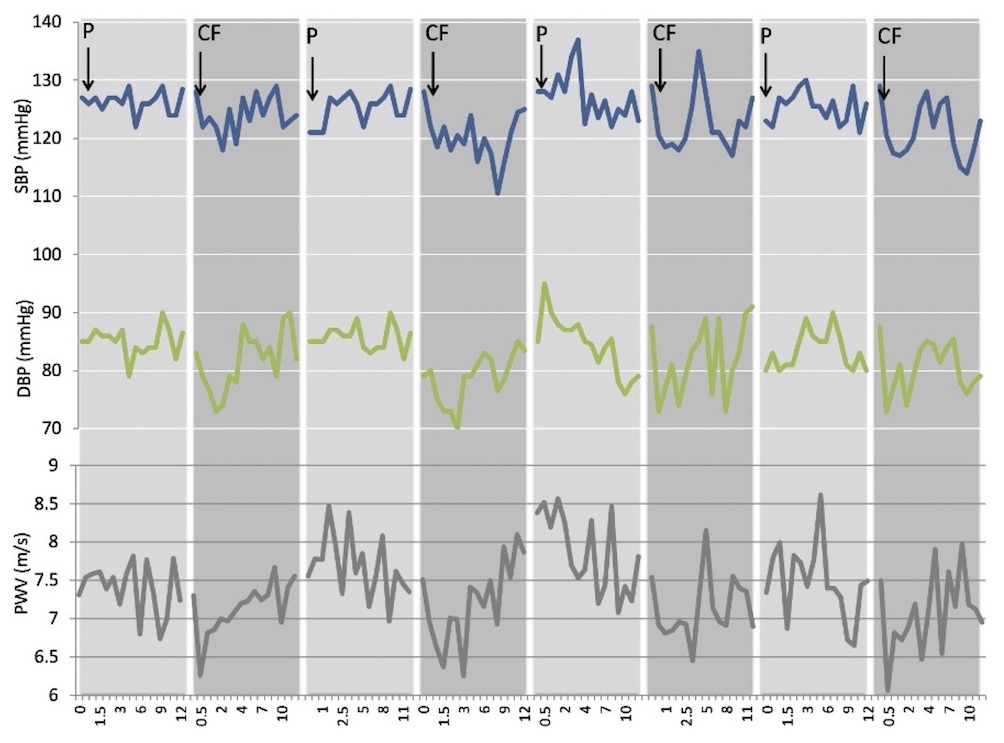


**Supplemental Figure 2**
